# Somatic cell fate maintenance in mouse fetal testes via autocrine/paracrine action of AMH and activin B

**DOI:** 10.1038/s41467-022-31486-y

**Published:** 2022-07-15

**Authors:** Karina F. Rodriguez, Paula R. Brown, Ciro M. Amato, Barbara Nicol, Chia-Feng Liu, Xin Xu, Humphrey Hung-Chang Yao

**Affiliations:** 1 Reproductive Developmental Biology Group, National Institute of Environmental Health Sciences, Durham, NC USA; 2 Lerner Research Institute, Cleveland Clinic, Cleveland, OH USA; 3 Epigenetics & Stem Cell Biology Laboratory, National Institute of Environmental Health Sciences, Durham, NC USA

**Keywords:** Embryology, Gonadal disorders, Reproductive biology, Differentiation, Transdifferentiation

## Abstract

Fate determination and maintenance of fetal testes in most mammals occur cell autonomously as a result of the action of key transcription factors in Sertoli cells. However, the cases of freemartin, where an XX twin develops testis structures under the influence of an XY twin, imply that hormonal factor(s) from the XY embryo contribute to sex reversal of the XX twin. Here we show that in mouse XY embryos, Sertoli cell-derived anti-Mullerian hormone (AMH) and activin B together maintain Sertoli cell identity. Sertoli cells in the gonadal poles of XY embryos lacking both AMH and activin B transdifferentiate into their female counterpart granulosa cells, leading to ovotestis formation. The ovotestes remain to adulthood and produce both sperm and oocytes, although there are few of the former and the latter fail to mature. Finally, the ability of XY mice to masculinize ovaries is lost in the absence of these two factors. These results provide insight into fate maintenance of fetal testes through the action of putative freemartin factors.

## Abstract (web summary)

Fate determination and maintenance of foetal testes in most animals occurs cell autonomously. Here the authors show in mouse XY embryos that Sertoli cell derived AMH and activin B together maintain Sertoli cell identity, and lack of both AMH and activin B leads to transdifferentiation into female granulosa cells.

## Introduction

The first morphogenetic event in sex determination is defined by the transformation of the gonadal primordium into a testis or an ovary. In most eutherian mammals, including humans and mice, the Y chromosome-linked transcription factor SRY steers the bipotential somatic cells toward the Sertoli cell lineage. Sertoli cells are responsible for testis morphogenesis, appearance of other somatic cell lineages, and differentiation of male germ cells^1,2^. In the absence of SRY, bipotential somatic cells differentiate into the ovarian granulosa cell lineage that express FOXL2. This lineage plasticity remains in Sertoli cells even when they become fully differentiated. DMRT1, also a transcription factor, is indispensable for the maintenance of Sertoli cell identity in adulthood. Without DMRT1, mature Sertoli cells transdifferentiate into FOXL2-positive granulosa cells^3,4,5^. These observations indicate that the initial establishment of the Sertoli cell lineage in the fetal testis depends on the transcriptional action of SRY while the maintenance of their identity in adulthood is dependent upon DMRT1 expression, both occurring in a cell-autonomously manner. However, this cell-autonomous mechanism of testis determination seems to be contradicted by the naturally occurring freemartin cases and experimental rodent models, which develop testis structures in the absence of Y chromosomes and SRY-dependent gene expression. In freemartin cases in sheep and cows, when the pregnant female carried XY and XX twins, the XX twin was often masculinized with the appearance of testicular structures in the ovary^6–13^. In experimental rodent models, when fetal ovaries were transplanted to an adult XY individual, the fetal ovaries lost their identity and became testis-like^14–16^. These observations suggest that hormone-like factor(s) produced in the XY individual could influence the somatic cell fate of the transplanted gonads.

Androgens and anti-Müllerian hormone (AMH) fit the profile of such factors because they are the hormones first produced by the fetal testis^17,18^. Androgens, despite their masculinizing effects on the male reproductive tract, do not have testis-inducing capacity as exposure of female embryos to androgens fail to induce testicular structures in the ovary of various species^19–23^. On the other hand, AMH induced freemartin effects on cultured sheep and rat ovaries^12,24,25^ and the AMH-AMH receptor system was identified as the master testis-determining gene in other species (reviewed in Pan et al.^26^). However, mice and humans lacking functional AMH had normal testis morphogenesis^27–29^ and fetal mouse ovaries cultured in the presence of AMH did not develop testicular cell markers^27^, suggesting other factors could compensate for its loss. We found that Sertoli cells in the mouse fetal testes express the gene inhibin beta B (*Inhbb*), whose protein products form the homodimers for activin B, a member of the transforming growth factor family, as is AMH^30^. The lack of expression of other inhibin beta subunits in fetal Sertoli cells indicate activin B as the main protein dimer produced by Sertoli cells. Similar to the *Amh* knockout mice, fetal testes of *Inhbb* knockout male developed normally despite minor vasculature defects^30^. To examine whether AMH and activin B could function synergistically or redundantly in facilitating testis morphogenesis, we generated mouse embryos lacking both *Amh* and *Inhbb* (*Amh*^−/−^; *Inhbb*^−/−^ or dKO hereafter) and examined the development of fetal testes.

Here, we show that mouse XY embryos lacking Sertoli cell-derived hormones AMH and activin B fail to maintain Sertoli cell fate at the gonadal poles, where Sertoli cells transdifferentiate into the female counterpart, granulosa cells, leading to ovotestis formation. The ovotestis remain to adulthood and are capable of producing both sperm and oocytes. Finally, the ability of XY mice to masculinize XX gonads is lost in the absence of these two factors.

## Results and discussion

### Loss of *Amh* and *Inhbb* results in XY ovotestis formation

**Fig. 1:**
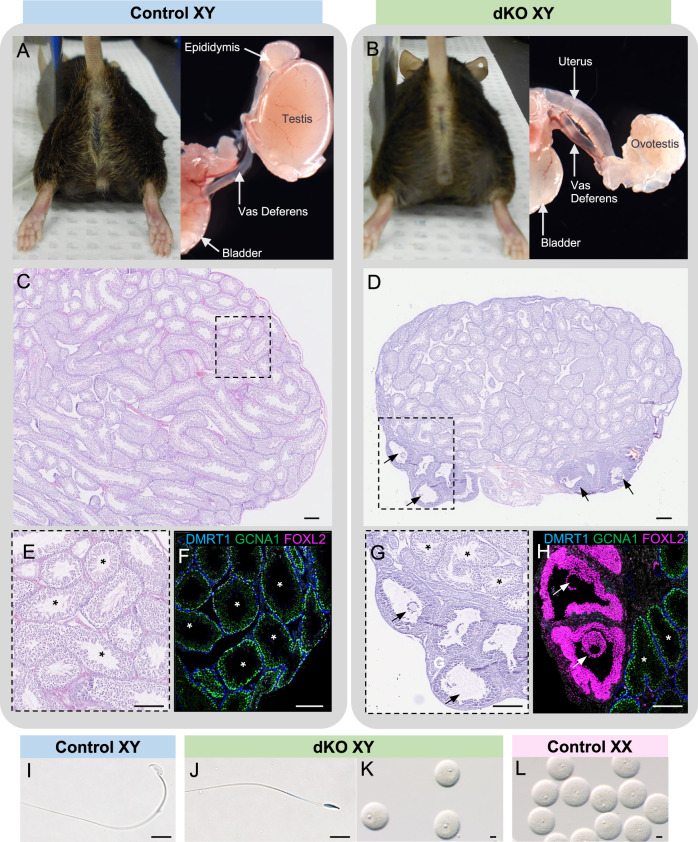
Loss of Amh/Inhbb in the XY mice leads to intersex phenotypes with ovotestis that produce oocytes and sperm in adulthood. **A**, **B** The external and internal genitalia of the five months old control and dKO XY mice. **C**, **D** Hematoxylin & Eosin staining of a section of control and dKO XY gonads at five months of age. Scale bar = 200 µm. **E**, **G** Higher magnification of the dotted lined area in **C**, **D**. **F**, **H** Immunofluorescence for DMRT1 (Sertoli cells, blue), GCNA1 (germ cells, green), and FOXL2 (granulosa cells, pink) in control and dKO XY gonads. Stars and arrows mark seminiferous tubules and follicles, respectively. Scale bar = 200 µm. **I**–**L** Sperm and oocytes were recovered from the control XY (sperm only, *n* = 2/2), control XX (oocytes only, *n* = 4/4) and dKO XY mice (both sperm and oocyte, *n* = 2/4) 46 h after PMSG stimulation. Scale bar = 25 µm.

Adult dKO XY mice had male external genitalia indistinguishable from the control XY mice (Fig. 1A, B). Internally, however, dKO XY mice developed as intersex with the presence of ovotestes and both male and female reproductive tracts (Fig. 1A, B and Sup Fig. 1). The ovotestes in the adult dKO XY mice were smaller than the testes of their wildtype littermates. In most cases, the ovotestes were cryptorchid, as a result of the presence of the female reproductive tract^28^. The ovotestis contained seminiferous tubules in the center and ovarian structures with follicles in the poles (Fig. 1D). The ovarian domains of the ovotestis contained follicles up to the preantral stages without the presence of corpus lutea, indicating that ovulation did not occur (Fig. 1G). The lineage marker for Sertoli cells (DMRT1) was properly detected in the seminiferous tubules of the control XY testis (Fig. 1F) and dKO ovotestis (Fig. 1H). The granulosa cell marker (FOXL2) was detected in the ovarian follicles of the ovotestis (Fig. 1H) but not in the control XY testis (Fig. 1F). To examine whether the follicles in the ovotestis can support oocyte development, we injected adult dKO XY and control XX mice with PMSG to induce follicular development and collected oocytes by mechanically disrupting the ovarian domains. Oocytes obtained from the ovotestis were morphologically comparable to those collected from control XX mice with the presence of germinal vesicles; however, they failed to mature in vitro (Fig. 1K, L). Spermatogenesis was incomplete in most seminiferous tubules of the ovotestis and few mature sperm were found in the epididymides of the dKO XY mice compared to their control XY littermates. These observations are likely caused by the dKO XY testes being cryptorchid (Fig. 1C–E, G, I, J).

### Initial testis formation is normal in *Amh/Inhbb* dKO mice

**Fig. 2:**
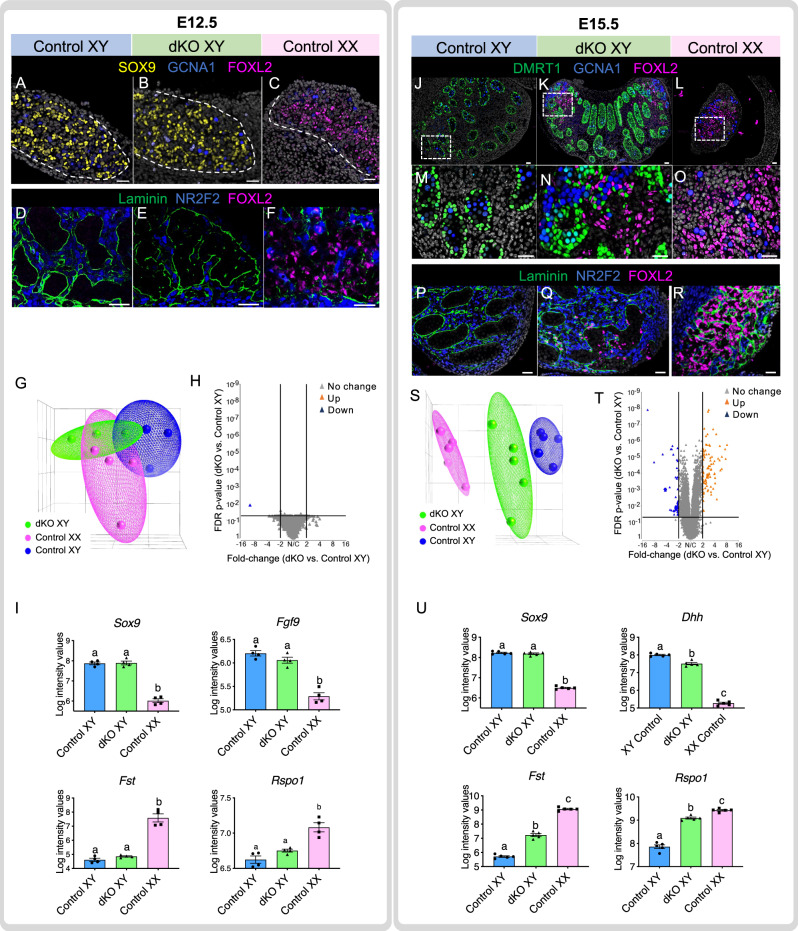
*Amh/Inhbb* dKO XY testis forms normally at E12.5 but becomes ovotestis by E15.5. **A**–**C** Immunofluorescence for SOX9 (Sertoli cells, yellow), GCNA1 (germ cells, blue), and FOXL2 (granulosa cells, pink) and **D**–**F** LAMININ (basal membrane, green), NR2F2 (interstitial cells, blue), and FOXL2 (granulosa cell, pink) on control XY (*n* = 4), dKO XY (*n* = 4), and control XX (*n* = 4) gonads at E12.5. **A**–**C** Dash lines highlight the boundary between the gonad and mesonephros. Scale bars = 25 um. **G** Principal Component Analysis (PCA) of the transcriptome of whole gonads at E12.5. **H** Volcano plot shows differentially expressed genes between the control XY and dKO gonads at E12.5 (2-fold, one-way ANOVA, FDR-corrected *p*-value < 0.05). **I** Relative mRNA expression level for genes critical for sex determination of the XY gonads (*Sox9* and *Fgf9*) and XX gonads (*Fst* and *Rspo1*), *N* = 4 per group. Different letters indicate statistical difference (one-way ANOVA, Tukey’s means separation test, *p* < 0.001). **J**–**O** Immunofluorescence for DMRT1 (Sertoli cells, green), GCNA1 (germ cells, blue), and FOXL2 (granulosa cells, pink). **M**–**O** higher magnification of inset shown in **J**–**L**. **P**–**R** LAMININ (basal membrane, green), NR2F2 (interstitial cells, blue), and FOXL2 (granulosa cells, pink) on control XY (*n* = 4), dKO XY (*n* = 4), and control XX gonads (*n* = 4) at E15.5. **S** Principal Component Analysis (PCA) of the transcriptome of whole gonads at E15.5. **T** Volcano plot shows differentially expressed genes between the control XY and dKO XY gonad at E15.5 (2-fold, one-way ANOVA, FDR-corrected *p*-value < 0.05). **U** Relative mRNA expression level for genes in the XY gonads (*Sox9* and *Dhh*) and XX gonads (*Fst* and *Rspo1)* *N* = 4 per group. Different letters indicate statistical difference (one-way ANOVA, Tukey’s means separation test, *p* < 0.001). **I**, **U** depict means ± SE of log intensity values. Source data are provided as a Source Data file. All data were collected from biologically independent samples.

We next investigated whether the partial sex-reversal phenotype in the dKO testis resulted from failure to initiate the molecular program for testis morphogenesis or to maintain the established testis fate. At embryonic day 12.5 (E12.5), one day after the initiation of testis morphogenesis, *Amh/Inhbb* dKO testes were morphologically indistinguishable from the wildtype testes. SOX9-positive Sertoli cells formed properly in both wildtype (Fig. 2A) and dKO testis (Fig. 2B). In addition, the basal membrane component laminin delineated the forming testis cords in both control and dKO testis with NR2F2 + cells in the interstitium (Fig. 2D, E). FOXL2, the lineage marker for granulosa cells, was absent in the testes of both the control and dKO XY gonad (Fig. 2A, B, D, E) at this stage while its expression was evident as expected in the control ovary (Fig. 2C, F). The global gene expression profiles between control and dKO XY gonads were statistically indistinguishable at E12.5 with similar clustering of individual samples among the three genotypes (control XY, dKO XY, and control XX gonads) (Fig. 2G, H, 2-fold, *p* < 0.05). Most importantly, the genes critical for Sertoli cell fate determination and differentiation (*Sox9* and *Fgf9*, Fig. 2I) and genes involved in ovarian fate determination (*Fst* and *Rspo1*) were not different between control XY and dKO XY gonads. This evidence indicated that testis determination and Sertoli cell fate specification were not compromised in the absence of AMH and activin B.

### Ovarian domains appear at the poles of *Amh*/*Inhbb* dKO testes

In contrast to the lack of effects at E12.5, morphological and molecular differences between the dKO and control XY gonads became apparent at E15.5 when the morphogenetic events were almost completed. The characteristic cord structure outlined by DMRT1-positive Sertoli cells in the control testis was mostly disintegrated in the poles of the dKO XY gonad (Fig. 2J, K, M, N). Cells positive for FOXL2 and negative for DMRT1 (Fig. 2K, N) appeared side-by-side, leading to a typical feature of an ovotestis^31^. The disintegration of testis cords in the poles of dKO XY gonad became apparent by the diminished laminin deposition and the appearance of FOXL2 + cells (Fig. 2P–R). Germ cells in the poles of the dKO XY gonad entered meiosis and became positive for the meiotic marker SYCP3 (comparable to the germs cells in the control ovary in Sup Fig. 2H, I). On the contrary, germ cells inside the cords at the center of the dKO gonads did not enter meiosis, comparable to the control testis (Sup Fig. 2F, G). Germ cells in the ovarian and testicular domains continued the program of folliculogenesis and spermatogenesis, respectively, into adulthood (Fig. 1D, G, H). Furthermore, the transcriptomic profile of the dKO XY gonads at E15.5 fell in between the control XY and XX transcriptomes (Fig. 2S). When comparing the transcriptome profiles between dKO and control XY gonads (Fig. 2T, 2-fold, *p* < 0.05), 98 genes were upregulated in the dKO testis. Among them, 88 (or 90%) were genes enriched in the ovary (Supp Fig. 3A), including ovarian somatic cell markers *Runx1, Rspo1, Foxl2,* and the meiosis marker *Stra8* (Fig. 2U and Supp Fig. 3C). On the other hand, 55 genes were downregulated in the dKO XY gonad. Among them, 48 (or 87%) were testis-enriched genes such as *Fshr, Serpina5, Ccl17 and Kazald1*. The transcriptional profile of genes markers for pre-Sertoli and Fetal Leydig cells (such as steroidogenic genes) between dKO and control XY testis was similar (Fig. 2U and Suppl Fig. 3C). These observations together indicate that the gonad in the *Amh/Inhbb* dKO male was committed to the testicular program at the beginning of testis morphogenesis (E12.5) but failed to maintain its identity as development continued (E15.5) as a result of the uprising ovarian program. These results implicate that AMH and activin B act synergistically in the fetal testis to suppress the ovarian program. The fact that complete XY to XX sex reversal did not occur in the absence of *Amh/Inhbb* suggests that other factors could compensate for the loss of these two factors, which both belong to the TGF-beta superfamily. Indeed, other TGF-beta ligands such as inhibin beta A (*Inhba*) and *Tgfb2* are expressed by various cell types in the XY gonads (Supp Fig. 4), which could have redundant roles as AMH and activin B.

### Femininization of supporting cells in the dKO testis

**Fig. 3:**
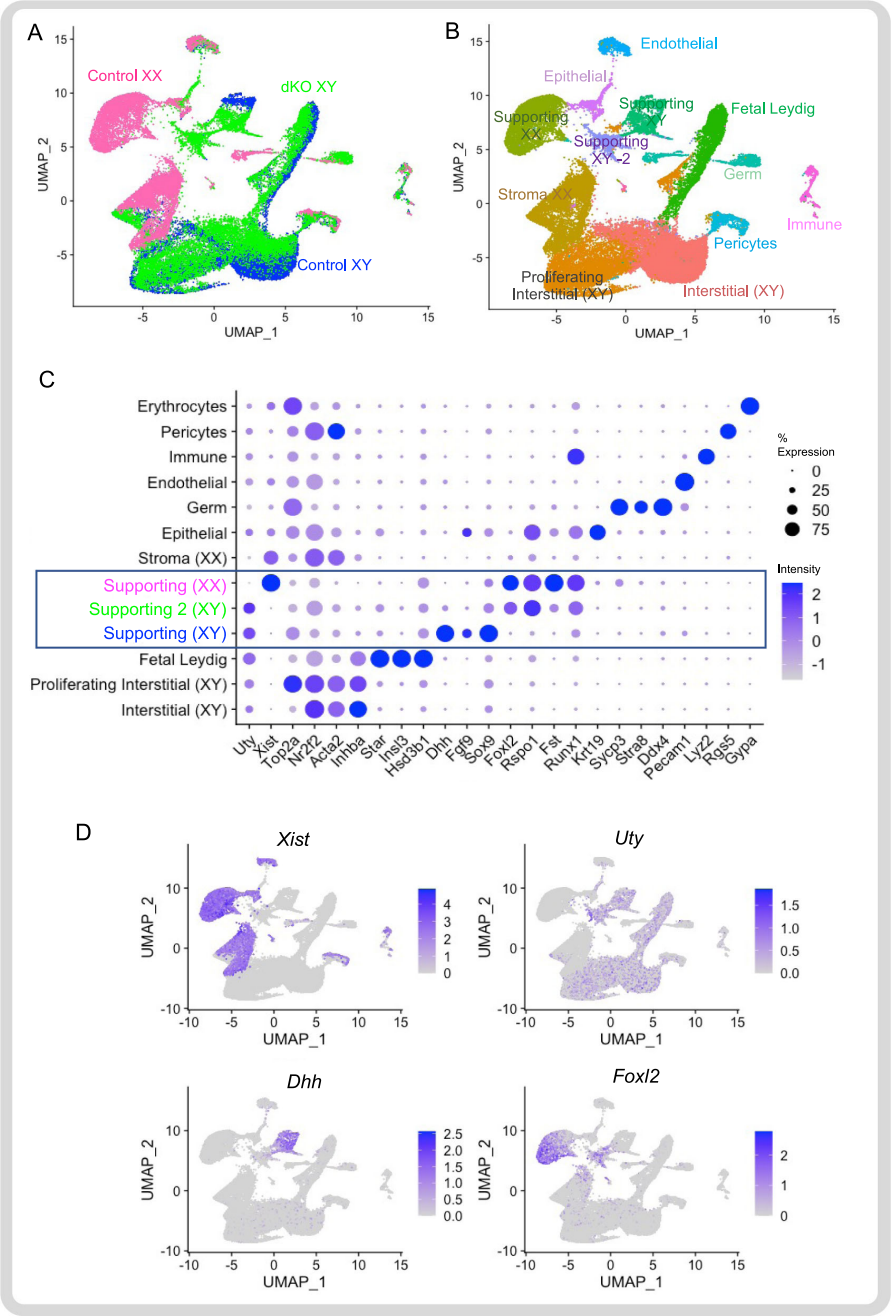
Single cell mRNA sequencing analysis of control XX (ovary), control XY (testis) and dKO XY (ovotestis) at E15.5. **A**, **B** Genotype distribution and cell type clustering based on unbiased clustering of transcriptomic similarity. Thirteen cell clusters were identified. **C** Dot plots of the expression of genes used to identify each cluster. The rectangle highlights both XY and XX supporting cell populations. **D** Cell type distribution of the expression of *Xist* (X or female marker), *Uty* (Y or male cell marker), Sertoli cell marker (*Dhh*) and granulosa cell marker (*FoxL2*). Source data are provided as a Source Data file. *N* = 2 biological independent samples per genotype.

**Fig. 4:**
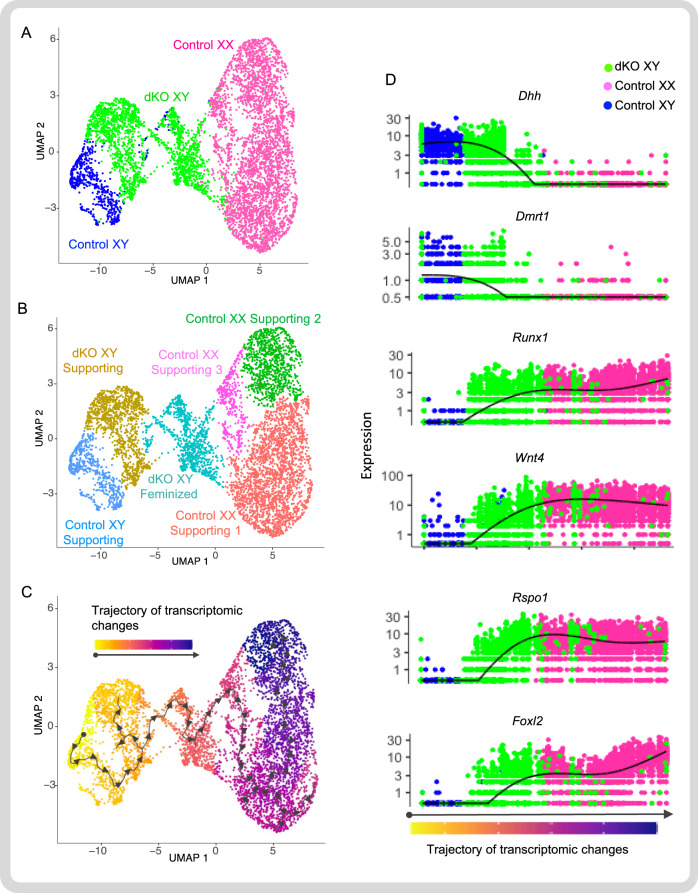
Feminization of the dKO XY supporting cells at the single cell level. **A** Single cell mRNA sequencing analysis and clustering of the supporting cells based on their genotypes: control XY (blue), dKO XY (green), and control XX (pink) gonads at E15.5. Each dot represents the transcriptome of a single cell. **B** Cell clustering based on their unique transcriptomes: one population in the XY control, two subpopulations in the dKO XY, and three subpopulations in the control XX supporting cell populations. **C** Trajectory of transcriptomic changes (pseudotime analysis) among supporting cells. Control XY supporting cell in the far left was arbitrarily designated as the starting point (black dot) of the differentiation. Arrows and the gradient of colors (yellow to dark blue) indicate the direction and progression of transcriptomic changes. **D** Transcriptome trajectory of six representative genes critical for sex determination of the XY gonads (*Dhh* and *Dmrt1*) and XX gonads (*Runx1*, *Wnt4*, *Rspo1*, and *Foxl2*). The X axis represents the arbitrary trajectory of transcriptome changes from a single control XY supporting cells toward control XX supporting cells (dark blue). Y axis indicates the gene expression level in each cell. The black lines represent the average expression of the genes over the progression. Source data are provided as a Source Data file. *N* = 2 biologically independent samples per genotype.

To gain insight into the testis-to-ovary populational shift of the dKO XY ovotestis and its potential underlying mechanism, we performed single cell mRNA sequencing on control XY, dKO XY, and control XX gonads at E15.5 (*n* = 2 per genotype). Using Uniform Manifold Approximation and Projection (UMAP) to unbiasedly distinguish and cluster cells with similar transcriptomic profiles, we analyzed a total of 39,350 cells from all 3 genotypes and their replicates. Based on the unique single cell sequencing barcodes for each genotype, we first separated the 39,350 cells based on their genotype (Fig. 3A). Specific cell types, including supporting cells (Sertoli in XY vs. granulosa in XX), interstitial cells, steroidogenic cells, germ cells, endothelial, epithelial, and immune cells (Fig. 3B, C & Supp Table 1), were further identified according to their unique transcriptomes and expression of known markers (Fig. 3D), demonstrating the validity of this approach. Next, we focused on the supporting cell lineages, which are the primary drivers for morphogenesis of the gonads, and analyzed them based on their distinct transcriptomes. The supporting cell lineages clustered into three populations based on their sexes and genotypes: control XY, dKO XY, and control XX (Fig. 4A). The control XY and XX supporting cells (Sertoli vs. granulosa) were distinctly separated from each other whereas dKO XY supporting cells were situated in between these two populations (Fig. 4A). Unbiased clustering further uncovered a single population in control XY supporting cells, three subpopulations in control XX supporting cells, and two subpopulations in dKO XY supporting cells (Fig. 4B). The first subpopulation in the dKO XY supporting cells had a transcriptomic profile similar to that of the control XY supporting cells (Fig. 4B and Sup Fig. 5B). The second subpopulation of dKO XY supporting cells, designated as “dKO XY feminized”, was the most deviated from the control XY supporting cells with a reduction in expression of control Sertoli cell genes (*Dhh*, *Sox9* and *Cst9)* and increased expression of granulosa cell markers (*Foxl2*, *Rspo1*, *Fst* and *Runx1*, Fig. 4D and Sup Fig. 5). This feminized subpopulation was also closest to the control XX supporting clusters (Fig. 4B, D), indicating that they were transitioning or transdifferentiating into XX supporting cells. This conclusion was supported by an analysis of the trajectory of transcriptomic changes (also known as pseudo-time analysis in Monocle^32–34^) among supporting cells of the three genotypes. When we arbitrarily designated the control XY supporting cells (the black dot in Fig. 4C) as the starting point, we observed a unique differentiation path or trajectory from the control XY towards dKO XY, and eventually ending as control XX supporting cells (Fig. 4C). When examining genes known for XY supporting cells (*Dhh* and *Dmrt1*) and XX supporting cells (*Runx1, Wnt4, Rspo1, and Foxl2*, Fig. 4D), we observed the unique transcriptomic trajectory with a decreasing trend of XY supporting cell genes and an increasing trend of XX supporting cell genes from the XY control, dKO XY, to the XX control supporting cells at the single cell level (Fig. 4D). Most notably, the increase in XX supporting gene expression in the dKO cells occurred prior to the decrease in XY supporting cell gene expression (Fig. 4D). These results support the model that in the absence of *Amh* and *Inhbb*, the XY supporting cell lineage lose their identity and gradually take on the characteristics of XX supporting cells. The appearance of the ovarian domain only in the poles, but not the center, of the dKO XY gonads is consistent with ovotestis phenotypes observed in mice^35–40^. In the case of the *Amh Inhbb* dKO XY, no differences in expression of genes involved in the initial testis differentiation (*Sox9, Fgf9, Fst, Rspo1*, Fig. 2I) were found, and the gonads differentiate normally in morphology, indicating that the feminization of the gonad occurs after *Sry/Sox9* expression. These observations support the hypothesis that at the center of the gonad the male supporting cells are further along the Sertoli cell fate than those at the poles.

### Maintenance of Sertoli cell identity by AMH and activin B

**Fig. 5:**
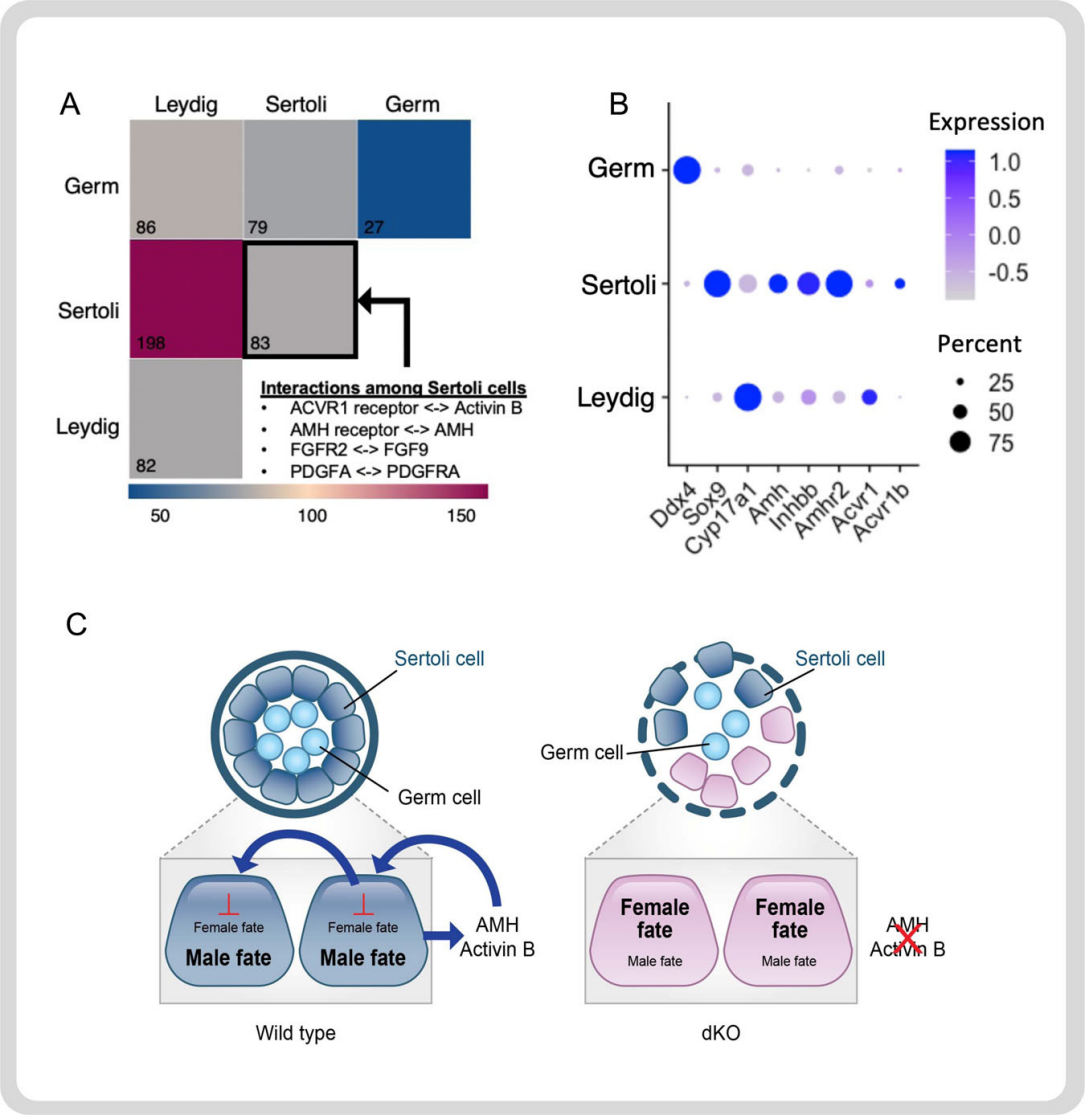
Autocrine/Paracrine action of AMH and activin B on Sertoli cells. **A** Statistical prediction of cell-cell communication via ligand and receptor interaction among germ cells, Sertoli cells, and Leydig cells from the single cell RNAseq dataset using CellPhoneDB. Each square in the 3 × 3 grid represents interaction between two cell types (germ vs germ, Sertoli vs Leydig, etc.). The number inside the square is the number of ligand/receptor pairs. Interactions for AMH, activin B and their receptors were significantly enriched among Sertoli cells (black outlined square; source data are provided as a Source Data file; *n* = 2 biologically independent samples per genotype). **B** Dot plots of the expression of *Amh*, *Inhbb*, and their receptors in germ cells, Sertoli cells, and Leydig cells. **C** Proposed model of the autocrine/paracrine action of AMH and Activin B. Sertoli cells secrete AMH and Activin B which act on an autocrine and paracrine way to block the female fate in the wild type Sertoli cells. In the absence of Amh and Activin B in the dKO, the cells become feminized at the testis poles.

We next investigated the mechanism of AMH/activin B action by examining the sources of ligands (AMH and activin B) and receptors (AMH receptors and activin receptors) in the wildtype fetal testis. We leveraged our single cell RNAseq dataset and interrogated such ligand/receptor interaction via CellPhoneDB, a statistical framework that predicts enriched cellular interactions between two cell types from single cell sequencing dataset^41^. We specifically compared the interaction among three cell types: germ cells, Sertoli cells, and Leydig cells (Fig. 5A). The 3 × 3 grid represents six different cell–cell interactions (germ cells vs germ cells, germ cells vs Leydig cells, etc.). The number in each square represents the number of ligand/receptor pairs. This unbiased analysis uncovered interactions between AMH/AMH receptor and activin/activin receptor that were significantly enriched among Sertoli cells. Indeed, expression of ligands (*Amh* and *Inhbb*) and their receptors (*Amhr2*, *Acvr1*, *Acvr1b*, and others) was enriched in Sertoli cell lineages (Fig. 5B and Sup Fig. 7). These data support the autocrine/paracrine nature of AMH and activin on Sertoli cells (Fig. 5C).

### Ability of AMH and activin B to masculinize fetal ovaries

**Fig. 6:**
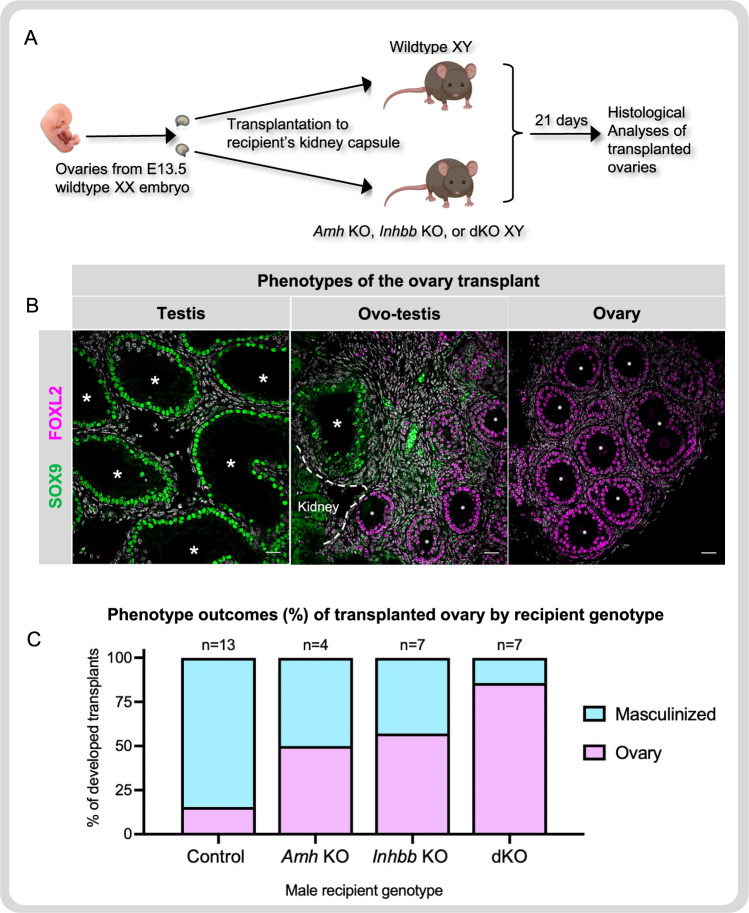
XY environment without Amh and Inhbb fails to masculinize the fetal ovary. **A** Mouse freemartin model was created by transplanting wildtype E13.5 ovaries under the kidney capsule of adult (five to seven months old) wild type, single *Amh*, single *Inhbb* KO, or dKO XY mice. Transplants were recovered 21 days after. **B** Immunofluorescence of Sertoli cell marker SOX9 (green) and granulosa cell marker FOXL2 (pink) was used to classify the transplanted tissue as testis (SOX9 + /FOXL2-), ovotestis (SOX9 + /FOXL2 + ), or ovary (SOX9-/FOXL2 + ). Asterisks and circles indicate seminiferous tubules and follicles, respectively. **C** The numbers and percentage of fetal ovary transplants that were masculinized with testis or ovotestis structure (blue) or remained as ovary (pink) in controls, *Amh* single knockout, *Inhbb* single knockout, or dKO XY recipients. All data from biologically independent samples. Scale bar = 25 µm. Source data are provided as a Source Data file. Mouse image was created with BioRender.com.

In freemartin cases in cattle and sheep, the testes of the male fetus secrete factors that masculinize the ovaries of the female fetus through the anastomosis of the blood vessels in the placenta (reviewed in Padula et al.^8^). Due to differences in placenta structures, freemartin cases were not reported in mice. Taketo et al.^16^ developed an alternative mouse model by transplanting fetal ovaries under the kidney capsule of recipient male mice to mimic the diffusion of the masculinizing factors from the recipient’s circulation onto the transplanted fetal ovaries. When E13.5 fetal mouse ovaries were transplanted under the kidney capsules of an adult wildtype male mouse, the majority of the fetal ovaries became ovotestes and, in a few cases, completely sex-reversed to testes after 21 days^15,16^. These observations indicate that hormone factors from adult wildtype male mice masculinized the transplanted fetal ovaries. Using this model, we examined whether AMH and activin B have the capacity to masculinize fetal ovaries in vivo (Fig. 6A). We first compared the serum level of AMH and activin B between wildtype and dKO adult XY mice and found the level became undetectable in the dKO XY male (Supplementary Fig. 6). When fetal ovaries were transplanted into control XY mice, 84.5% (11 out of 13) of the transplants became masculinized (expressing SOX9) and 15.4% (2 out of 13) remained as ovaries (expressing FOXL2 and no Sox9, Fig. 6A–C). These numbers were similar to published results^15,16^. In contrast, the adult dKO recipient males lost their ability to masculinize the fetal ovary with 85.7% (6 out of 7) of the transplanted ovaries remaining as ovaries while only 1 (14%) was masculinized. The masculinizing ability of *Amh* and *Inhbb* single knockout male recipients fell between the wildtype and dKO male recipients. These observations support the hypothesis that AMH and activin B have an additive ability to masculinize the fetal ovaries.

In summary, we identified a mechanism in fetal Sertoli cells that maintains their identity. Sertoli cell-derived AMH and activin B act as autocrine and paracrine factors on Sertoli cells, which express their receptors. These two factors maintain the testis program in Sertoli cells and when both are absent, the granulosa cell program emerges which leads to partial sex reversal. The incomplete sex reversal of the *Amh/Inhbb* double knockout testis (ovotestis structure and feminized transcriptomes and cell populations) further indicates that other factors, such as other TGF-beta proteins^27,42–45^, could compensate for the loss of these two factors. The structure of ovotestis in the dKO male resembles that of other ovotestis cases^35,39^; however, the forming process of such structure is distinctly different. The ovotestis in most cases is established at the onset of testis determination in the XY mouse embryos with the central part of the gonad following the testicular program and the flanking poles developing into an ovarian structure simultaneously^38,46^. In the absence of *Amh* and *Inhbb*, on the other hand, the fetal testis formed properly in the beginning, and the ovarian domains only arose later when Sertoli cells transdifferentiated into their female counter parts. Most intriguingly, in contrast to most ovotestis cases that disappear before birth, the dKO ovotestes were maintained into adulthood and were capable of sustaining spermatogenesis and folliculogenesis. Finally, the results of transplantation experiments support that in addition to their autocrine/paracrine properties on fetal Sertoli cells, AMH and activin B have the endocrine property of the freemartin factors that can masculinize the fetal ovary. These results not only advance our understanding of the process of gonadal sex differentiation, but also provide insight into the mechanisms behind cases of ovotestis and disorders of sex development in humans.

## Methods

### Mouse models

Wildtype and double knockout of *Amh* and *Inhbb* mice were generated from crossing the single heterozygous *Amh*^−/+^ (Jax 002188) and *Inhbb*^+/−^ (Jax 002442) in C57BL/6 background. Female mice were timed-mated and the day of detection of vaginal plug was considered embryonic day or E0.5. Mice were housed on a 12 h light:dark cycle, temperature range 70–74 °F and relative humidity range from 40 to 50%. All animal studies were conducted in accordance with the NIH Guide for the Care and Use of Laboratory Animals and approved by the National Institute of Environmental Health Sciences (NIEHS) Animal Care and Use Committee.

### RNA extraction, microarray and data analysis

On either E12.5 or E15.5, dams were euthanized, and fetal gonads (separated from the mesonephros) were collected, snap-frozen and stored at −80 °C. Genotyping was performed and RNA from gonads from *Amh*^−/−^; *Inhbb*^−/−^ (dKO XY), *Amh*^+/+^; *Inhbb*^+/+^ (Control XY and XX; *n* = 4 from E12.5 and *n* = 5 at E15.5 were isolated). Gene expression analysis was conducted using Affymetrix Mouse Transcriptome Clariom D arrays (Affymetrix, Santa Clara, CA). One hundred ng of total RNA was amplified as directed in the Nugen WT-Ovation Pico RNA Amplification System protocol and labeling with biotin following the Nugen Encore Biotin Module protocol. Five and one-half micrograms (5.5 μg) of amplified biotin-cDNAs were fragmented and hybridized to each array for 16 h at 45 °C in a rotating hybridization oven. Array slides were stained with streptavidin/phycoerythrin utilizing a double-antibody staining procedure and then washed for antibody amplification per the GeneChip Hybridization, Wash and Stain Kit and user manual following protocol FS450-0001. Arrays were scanned in an Affymetrix Scanner 3000 and data was obtained using TAC Software. Gene expression analyses were conducted with Partek software (Partek Genomics Suite v 7.19.1125, St. Louis, Missouri) using a one-way ANOVA comparing the RMA normalized log2 intensities.

### Single cell RNA sequencing

Fetal gonads were collected at E15.5. Gonads were isolated in PBS + 0.04% BSA and gonad pairs from the same animals were pooled and dissociated for 30 min at 37 C in dissociation buffer (1.2 U/ml Dispase II, 1 mg/ml collagenase B, 5 U/ml Dnase I in PBS). Cells were washed, counted and frozen at −80 °C in 50% FBS, 10% DSMO in DMEM/F12 and transferred to liquid nitrogen storage. Once genotyping was determined, single cell suspension from wild type control females (Control XX), wild type control males (Control XY) and *Amh*^−/−^; *Inhbb*^−/−^ (dKO XY) were thawed for 2 min at 37 °C, washed and cells were resuspended in PBS + 0.04% BSA. The 10× Genomics protocol was used for cell capture and library preparation. The resulting library was sequenced using an Illumina NovaSeq instrument (two biological replicates per genotype).

### Data processing and analysis for single cell RNA sequencing

Cell Ranger (v3.0) was utilized for count, alignment, filtering, cell barcode and UMI counting. Barcode swapping correction was performed^47^. FASTQ files of the corrected cell count matrixes were generated. The data were analyzed using Seurat^48^ package (v.3.2.2) on RStudio (v. 1.3.1073). Code used for analysis is included in the Source Data file. Seurat objects of each biological replicates were created (min.cells = 10, min.features = 400) and each biological replicate was combined using the “merge” function. A subset of the data was created using the following cutoffs: nFeature_RNA > 2500 & nFeature_RNA < 9000 & percent.mt < 25 & percent.Hbb < 20. The data was then normalized using the “NormalizeData” function (scale.factor = 10000). A linear model was applied to the data using the “Scaledata” command. PCA was run on the scaled data and visualization was done using UMAP with dims = 20. Differentially expressed marker genes in each cluster were found using FindAllMarkers function based on the Wilcoxon rank-sum test. Cluster identification was done according to established cell markers. Reclustering of male and female supporting cells as well as the feminized cluster was done using the “Subset” command, with data normalized and scaled, sorting out cells positive for *Sycp3* and *Tcf21* (germ and interstitial cell markers respectively). PCA was run on the scaled data and visualization was done using UMAP with dims = 8. Numbers of cells per cluster are listed in Sup. Tables 1 and 2. A Seurat object from the reclustered cells was created and loaded into Monocle3 for pseudotime analysis to determine differentiation trajectory using Control XY supporting cells as time 0 (T0) in pseudotime^32–34^. CellPhoneDB(https://www.cellphonedb.org)^41^, was used to predict cell-cell communication based on ligand-receptor interactions. Normalized counts were extracted from the Seurat object. Significant ligand-receptor pairs were called independently for wildtype males and dKO Germ, Supporting and Leydig cells respectively. The dotblot function in CellphoneDB was used to plot the data.

### Superovulation experiment

Adult control XX (*n* = 4), control XY (*n* = 2), and dKO XY (*n* = 4; 3–6 months old) were injected intra-peritoneally with 5 IU of PMSG and euthanized using C02 46 h after injection. Gonads were isolated, washed, and mechanically dissociated using 15 Ga needles to extract oocytes. Epididymides of both XY controls and dKO males were isolated and mechanically dissociated for sperm isolation.

### Ovary transplantation experiments

Fetal ovaries from wildtype C57BL/6 embryos were harvested at day E13.5. The ovaries were transferred to droplets of DMEM/F-12 media with 15 mM HEPES (Thermo Fisher Scientific cat. #11330032) supplemented with penicillin/streptomycin (Sigma-Aldrich cat. #P0781) and kept at 37 °C on a warming tray. Male recipients (control, *Amh* KO, *Inhbb* KO or dKO), between 12 and 32 weeks old with an average age of 17.6 weeks received one fetal ovary per kidney under the capsule. Twenty-one days after the transplantation, the transplanted ovaries were recovered and fixed in 10% neutral buffered formalin overnight at 4 °C. The tissues were washed and embedded in paraffin for immunofluorescence (see below).

### Hormone measurements

Serum samples from adult (4–6 months of age) XY controls (*n* = 6) and dKO XY (*n* = 8) were collected and stored at −80 until analysis was performed. Serum hormone levels for AMH (Ansh Labs, AL-113, intra assay CV = 3.6%, reportable range = 4.06–260 ng/mL), INHBB (Ansh Labs, AL-156, intra assay CV = 7.1%. reportable range = 7.3–1163.0 pg/mL) and Testosterone (IBL IB79106, intra assay CV = 5.4%, reportable range = 10.0–1600.0 ng/dL) were measured at the University of Virginia Center for Research in Reproduction Ligand Assay and Analysis Core by ELISA.

### Immunofluorescence

Gonads were collected and fixed in 4% PFA for 1 h at room temperature, washed in PBS and stored at 4 °C and either were imbedded in paraffin and sectioned (5 µm) or placed in MeOH for wholemounts. Following deparaffinization, sections were rehydrated in decreasing alcohol gradients and antigen retrieval was performed in 0.1 mM citric acid (Vector Labs) for 20 min in the microwave and then cooled down to room temperature. All samples were blocked in PBS-Triton X-100 solution with 5% normal donkey serum for 1 h and incubated with primary antibodies at 4 °C overnight. Primary antibodies used included (previously optimized in^40,49,50^: Anti-DMRT1 (1:500; kindly provided by David Zarkower from University of Minnesota), FOXL2 (1:200, Novus Biologicals cat. #NB100-1277), LAMININ (1:300; Sigma #L9393), GCNA1 (1:1000, Abcam # ab82527), SYCP3 (1:300, Abcam # ab15093), SOX9 (1:300, TransGenic #KO608), NR2F2 (1:300, R&D Systems # PP-H7147-00) and AMH (1:500, Santa Cruz # sc-6886). Secondary antibodies were used at 1:200 dilution (Invitrogen/Life Technology): Donkey anti-rabbit Alexa 488 (A21206), Donkey anti-mouse Alexa 568 (A10037), Donkey anti-rat Alexa 594 (A21209), Donkey anti-goat Alexa 647 (A21447). Imagining for wholemounts was done on a Zeiss LSM 880 confocal microscope using Zen software. Imaging for sections was performed on a Leica DMI4000 SPE confocal microscope using Leica Application Suite X (LAS X) software.

### Statistical analyses

No sample size calculation was performed. All histological experiments were repeated at least 4 times with independent biological replicates with similar results. Microarray analysis for both time points analyzed, E12.5 and E15.5, were performed independently. *N* = 4 (E12.5) and *N* = 5 (E15.5) biological replicates per genotype were used, respectively. ANOVA was performed and differential gene expression was determined by comparing the gene expression profile between the dKO XY and Control XY using 2-fold change and *p* < 0.05 with FDR correction. Raw log2 intensity data were analyzed using Prism, version 6 (GraphPad Software, La Jolla, CA), analyzed by one -way ANOVA and means separated by Tukey’s multiple comparisons test. Graphs show mean ± SEM.

### Reporting summary

Further information on research design is available in the Nature Research Reporting Summary linked to this article.

## Supplementary information

Supplementary Information

Reporting Summary

## Data Availability

The microarray and scRNAseq data generated in this study have been deposited in the GEO database. The GEO accession numbers for the data sets are: E12.5 microarray, GSE196826; E15.5 microarray, GSE196841 and E15.5 scRNAseq, GSE19697. The data sets used for the CellphoneDB analysis are listed in [https://www.cellphonedb.org/downloads]. Source data and code used for scRNAseq analysis (in Source Data file) are provided with this paper. Source data are provided with this paper.

## Source data

Source Data

## Code availability

Code used in the analysis of scRNAseq data is provided under supplementary information.

## References

[1] Gonen N, Lovell-Badge R (2019). The regulation of Sox9 expression in the gonad. Curr. Top. Dev. Biol..

[2] Ungewitter EK, Yao HH (2013). How to make a gonad: cellular mechanisms governing formation of the testes and ovaries. Sex. Dev..

[3] Matson CK (2011). DMRT1 prevents female reprogramming in the postnatal mammalian testis. Nature.

[4] Minkina A (2014). DMRT1 protects male gonadal cells from retinoid-dependent sexual transdifferentiation. Dev. Cell.

[5] Kim S, Bardwell VJ, Zarkower D (2007). Cell type-autonomous and non-autonomous requirements for Dmrt1 in postnatal testis differentiation. Dev. Biol..

[6] Ewen AH, Hummason FA (1947). An ovine freemartin. J. Hered..

[7] Forbes TR (1946). The origin of freemartin. Bull. Hist. Med..

[8] Padula AM (2005). The freemartin syndrome: an update. Anim. Reprod. Sci..

[9] Remnant JG (2014). Novel gonadal characteristics in an aged bovine freemartin. Anim. Reprod. Sci..

[10] Smith MC, Dunn HO (1981). Freemartin condition in a goat. J. Am. Vet. Med. Assoc..

[11] Vigier B, Tran D, Legeai L, Bezard J, Josso N (1984). Origin of anti-Mullerian hormone in bovine freemartin fetuses. J. Reprod. Fertil..

[12] Vigier B, Watrin F, Magre S, Tran D, Josso N (1987). Purified bovine AMH induces a characteristic freemartin effect in fetal rat prospective ovaries exposed to it in vitro. Development.

[13] Wilkes PR, Munro IB, Wijeratne WV (1978). Studies on a sheep freemartin. Vet. Rec..

[14] Taketo T, Koide SS, Merchant-Larios H (1984). Induction of testicular development in the fetal mouse ovary. Ann. N. Y Acad. Sci..

[15] Taketo T, Merchant-Larios H (1986). Gonadal sex reversal of fetal mouse ovaries following transplantation into adult mice. Prog. Clin. Biol. Res..

[16] Taketo T, Merchant-Larios H, Koide SS (1984). Induction of testicular differentiation in the fetal mouse ovary by transplantation into adult male mice. Proc. Soc. Exp. Biol. Med..

[17] Wilhelm D, Palmer S, Koopman P (2007). Sex determination and gonadal development in mammals. Physiol. Rev..

[18] Swain A, Lovell-Badge R (1999). Mammalian sex determination: a molecular drama. Genes Dev..

[19] Zhou, Y. et al. Maternal testosterone excess contributes to reproductive system dysfunction of female offspring mice. *Endocrinology* **161**, 10.1210/endocr/bqz011 (2020). 10.1210/endocr/bqz011 31680156

[20] Saloniemi T (2007). Activation of androgens by hydroxysteroid (17beta) dehydrogenase 1 in vivo as a cause of prenatal masculinization and ovarian benign serous cystadenomas. Mol. Endocrinol..

[21] Barsky M (2021). Fetal programming of polycystic ovary syndrome: effects of androgen exposure on prenatal ovarian development. J. Steroid Biochem. Mol. Biol..

[22] Abbott DH, Barnett DK, Bruns CM, Dumesic DA (2005). Androgen excess fetal programming of female reproduction: a developmental aetiology for polycystic ovary syndrome?. Hum. Reprod. Update.

[23] Steckler T, Wang J, Bartol FF, Roy SK, Padmanabhan V (2005). Fetal programming: prenatal testosterone treatment causes intrauterine growth retardation, reduces ovarian reserve and increases ovarian follicular recruitment. Endocrinology.

[24] Vigier B (1988). Anti-mullerian hormone and freemartinism: inhibition of germ cell development and induction of seminiferous cord-like structures in rat fetal ovaries exposed in vitro to purified bovine AMH. Reprod. Nutr. Dev..

[25] Vigier B, Magre S, Charpentier G, Bezard J, Josso N (1991). Anti-mullerian hormone and natural and experimental freemartin effect. Bull. Assoc. Anat. (Nancy).

[26] Pan Q (2021). Evolution of master sex determiners: TGF-beta signalling pathways at regulatory crossroads. Philos. Trans. R. Soc. Lond. B Biol. Sci..

[27] Ross AJ, Tilman C, Yao H, MacLaughlin D, Capel B (2003). AMH induces mesonephric cell migration in XX gonads. Mol. Cell Endocrinol..

[28] Behringer RR, Finegold MJ, Cate RL (1994). Mullerian-inhibiting substance function during mammalian sexual development. Cell.

[29] Josso N, Belville C, di Clemente N, Picard JY (2005). AMH and AMH receptor defects in persistent Mullerian duct syndrome. Hum. Reprod. Update.

[30] Yao HH, Aardema J, Holthusen K (2006). Sexually dimorphic regulation of inhibin beta B in establishing gonadal vasculature in mice. Biol. Reprod..

[31] Koopman P, Wilhelm D (2011). Insights into the aetiology of ovotesticular DSD from studies of mouse ovotestes. Adv. Exp. Med Biol..

[32] Cao J (2019). The single-cell transcriptional landscape of mammalian organogenesis. Nature.

[33] Qiu X (2017). Reversed graph embedding resolves complex single-cell trajectories. Nat. Methods.

[34] Trapnell C (2014). The dynamics and regulators of cell fate decisions are revealed by pseudotemporal ordering of single cells. Nat. Biotechnol..

[35] Ludbrook LM (2012). Excess DAX1 leads to XY ovotesticular disorder of sex development (DSD) in mice by inhibiting steroidogenic factor-1 (SF1) activation of the testis enhancer of SRY-box-9 (Sox9). Endocrinology.

[36] Wilhelm D (2009). Antagonism of the testis- and ovary-determining pathways during ovotestis development in mice. Mech. Dev..

[37] Bagheri-Fam S (2008). Loss of Fgfr2 leads to partial XY sex reversal. Dev. Biol..

[38] Bogani D (2009). Loss of mitogen-activated protein kinase kinase kinase 4 (MAP3K4) reveals a requirement for MAPK signalling in mouse sex determination. PLoS Biol..

[39] Eicher EM, Shown EP, Washburn LL (1995). Sex reversal in C57BL/6J-YPOS mice corrected by a Sry transgene. Philos. Trans. R. Soc. Lond. B Biol. Sci..

[40] Nicol B (2018). Genome-wide identification of FOXL2 binding and characterization of FOXL2 feminizing action in the fetal gonads. Hum. Mol. Genet.

[41] Efremova M, Vento-Tormo M, Teichmann SA, Vento-Tormo R (2020). CellPhoneDB: inferring cell-cell communication from combined expression of multi-subunit ligand-receptor complexes. Nat. Protoc..

[42] Cai K (2011). Action mechanism of inhibin alpha-subunit on the development of Sertoli cells and first wave of spermatogenesis in mice. PLoS ONE.

[43] Miles DC (2013). Signaling through the TGF beta-activin receptors ALK4/5/7 regulates testis formation and male germ cell development. PLoS ONE.

[44] Memon MA, Anway MD, Covert TR, Uzumcu M, Skinner MK (2008). Transforming growth factor beta (TGFbeta1, TGFbeta2 and TGFbeta3) null-mutant phenotypes in embryonic gonadal development. Mol. Cell Endocrinol..

[45] Miura K (2019). Molecular and genetic characterization of partial masculinization in embryonic ovaries grafted into male nude mice. PLoS ONE.

[46] Colvin JS, Green RP, Schmahl J, Capel B, Ornitz DM (2001). Male-to-female sex reversal in mice lacking fibroblast growth factor 9. Cell.

[47] Griffiths, J. A., Richard A. C., Bach K., Lun A. T. L. & Marioni J. C. Detection and removal of barcode swapping in single-cell RNA-seq data. *Nat. Commun.* **9**, 2667 (2018). 10.1038/s41467-018-05083-x PMC6039488 29991676

[48] Satija R, Farrell JA, Gennert D, Schier AF, Regev A (2015). Spatial reconstruction of single-cell gene expression data. Nat. Biotechnol..

[49] Nicol B, Rodriguez K, Yao HH (2020). Aberrant and constitutive expression of FOXL2 impairs ovarian development and functions in mice. Biol. Reprod..

[50] Nicol, B. et al. RUNX1 maintains the identity of the fetal ovary through an interplay with FOXL2. *Nat. Commun.* **10**, 5116 (2019). 10.1038/s41467-019-13060-1 PMC6848188 31712577

