# Supplementary material for: Somatic cell fate maintenance in mouse fetal testes via autocrine/paracrine action of AMH and activin B
Source: Nat Commun. 2022 Jul 15;13:4130. doi: 10.1038/s41467-022-31486-y (PMC9287316; doi:10.1038/s41467-022-31486-y)
Supplement: Supplementary file 1 — Supplementary Information [file 41467_2022_31486_MOESM1_ESM.pdf]

## Supplemental Table 1

| | Control XY | dKO XY | Control XX |
|---|---|---|---|
| Interstitial (XY) | 7377 | 3438 | 4 |
| Proliferating Interstitial (XY) | 3724 | 3347 | 9 |
| Fetal Leydig | 1868 | 1423 | 0 |
| Supporting (XY) | 761 | 1205 | 0 |
| Supporting (XY) 2 | 34 | 899 | 8 |
| Supporting (XX) | 0 | 93 | 4173 |
| Stroma (XX) | 520 | 989 | 4470 |
| Epithelial | 84 | 84 | 342 |
| Germ | 24 | 695 | 485 |
| Endothelial | 242 | 376 | 339 |
| Immune | 258 | 286 | 81 |
| Pericytes | 481 | 531 | 171 |
| Erythrocytes | 14 | 32 | 29 |
| Total cells | **15387** | **13852** | **10111** |

## Supplemental Table 2

| | Control XY | dKO XY | Control XX |
|---|---|---|---|
| XX Supporting 1 | 0 | 51 | 2254 |
| XX Supporting 2 | 0 | 4 | 976 |
| XX Supporting 3 | 2 | 19 | 403 |
| dKO Feminized | 33 | 827 | 15 |
| XY Supporting 1 | 565 | 6 | 0 |
| XY Supporting 2 | 33 | 1108 | 0 |
| Total cells | **633** | **2015** | **3648** |

**Supplemental Figure 1**

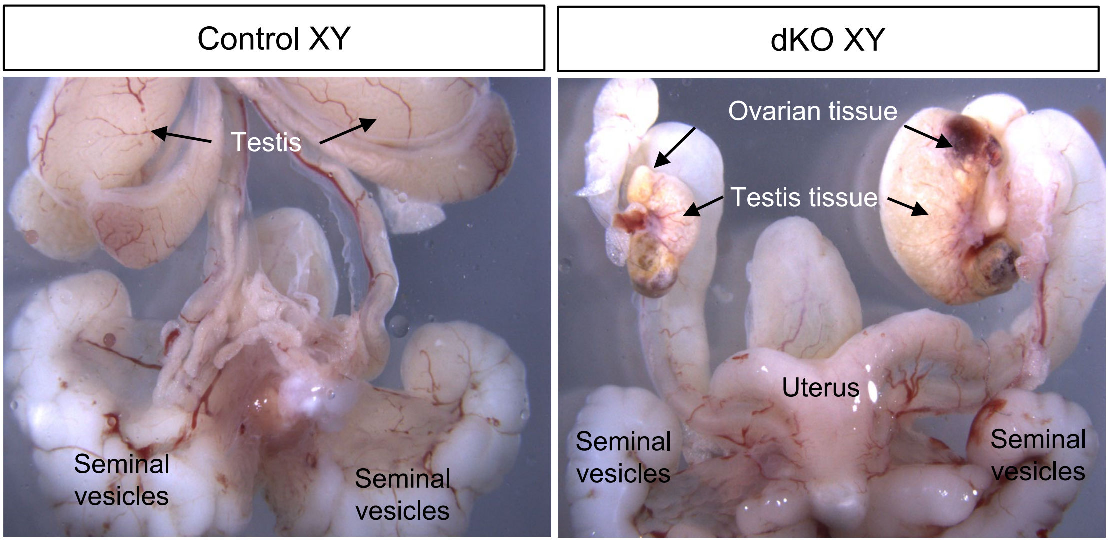


Supplemental Figure 1: Reproductive tracts from control XY and dKO XY adult mice. The dKO XY had the expected male reproductive organs such as seminal vesicles and epididymides; however, they also retained female reproductive organs including uterus. Ovarian tissues were visibly present along with the testis tissue. Variability on the size of the ovotestis was observed in adult dKO animals (n=12).

**Supplemental Figure 2**

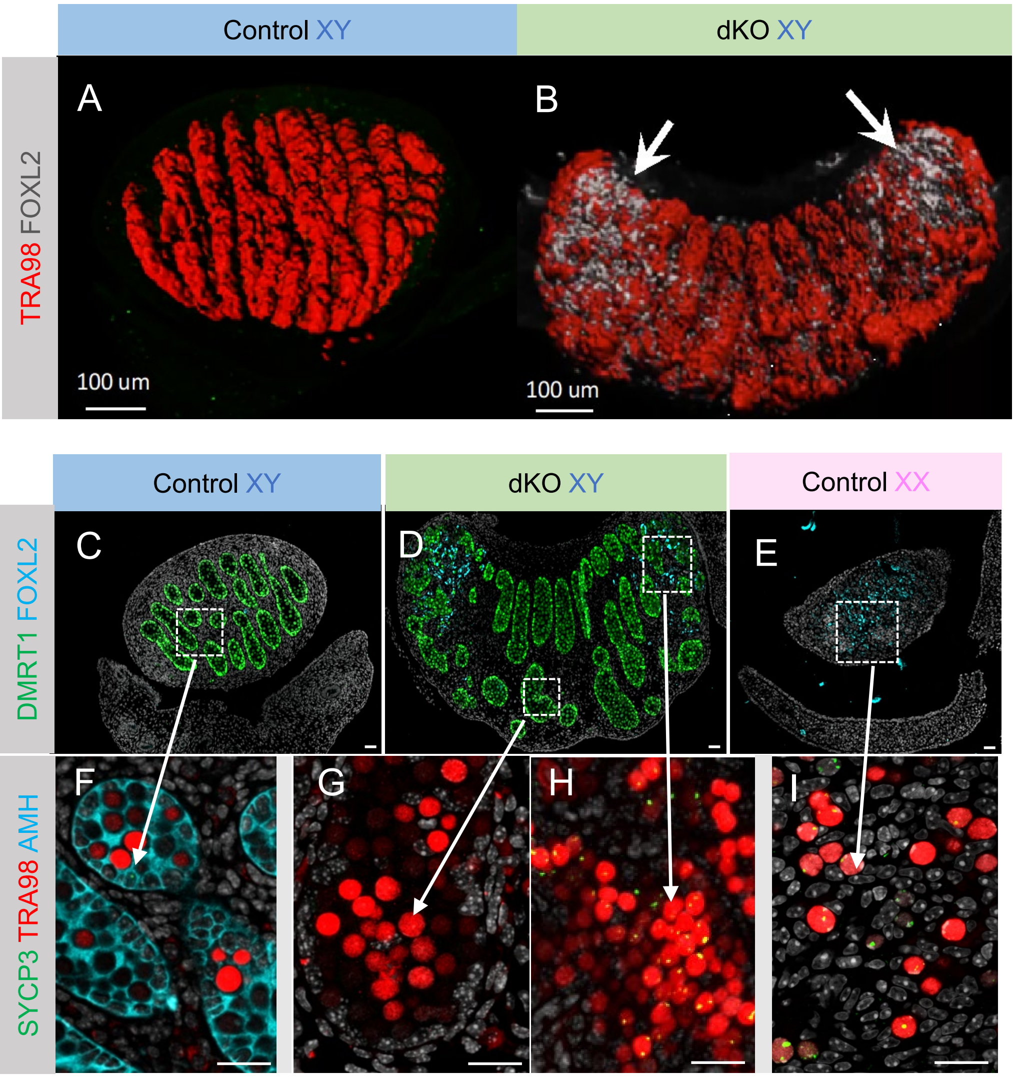


Supplemental Figure 2: (A-B) Immunofluorescence for TRA98 and FOXL2 in control and dKO XY gonads at E15.5 Arrows indicate the ovarian structure in the poles. (C-I) ) Immunofluorescence for DMRT1, FOXL2, SYCP3, TRA98, and AMH in control XY, dKO XY, and control XX gonads at E15.5. Scale bar = 25 um.

**Supplemental Figure 3**

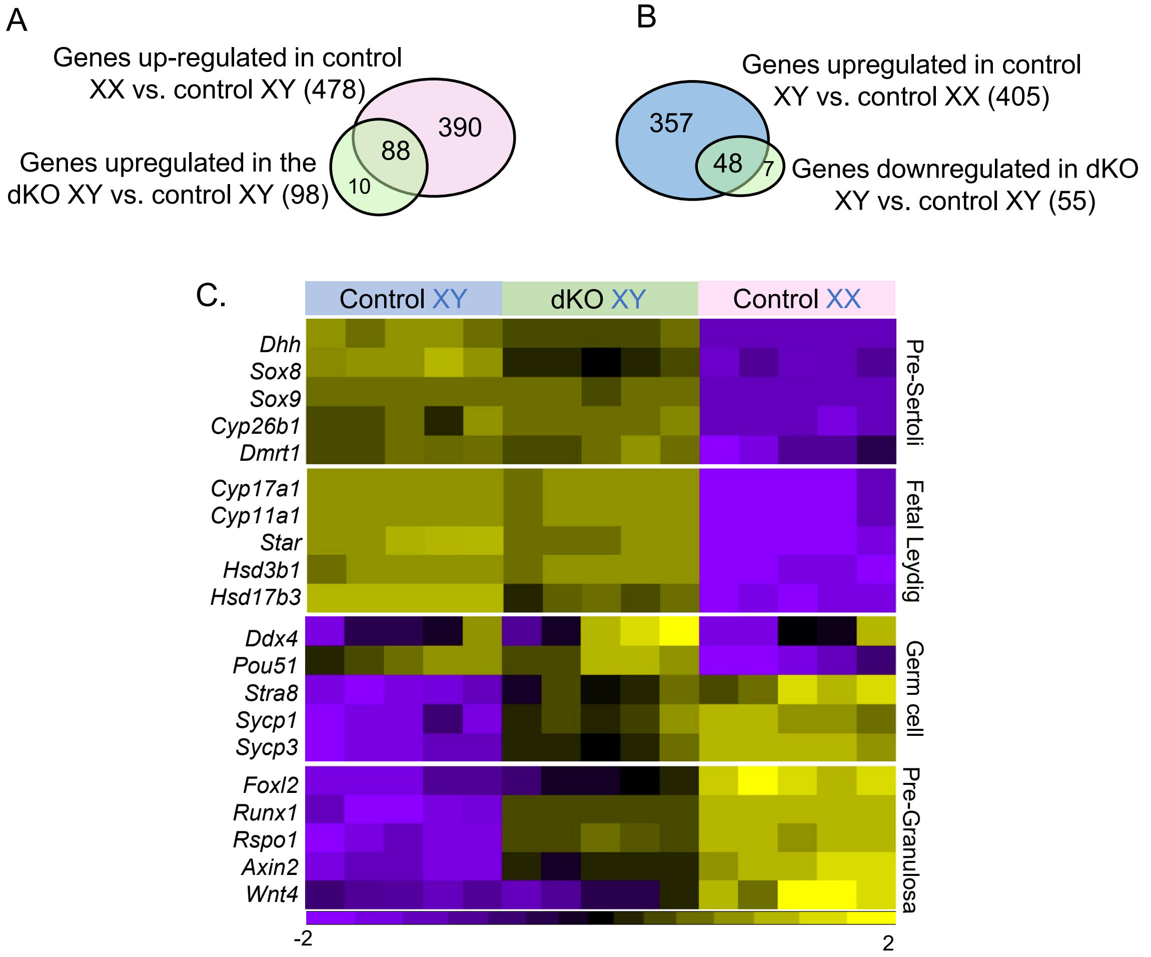


Supplemental Figure 3: (A-B) Transcriptome analysis of control XY, dKO XY, and control XX gonads at E15.5 (Source data are provided as a Source Data file; one-way ANOVA, p<0.05 FDR). (C) Heat maps of genes critical for sex determination and gonadal development in control XY, dKO XY, and control XX gonads at E15.5. Each column represents the expression of a single gonad. Yellow and purple indicate up-regulation and down-regulation, respectively.

**Supplemental Figure 4**

A

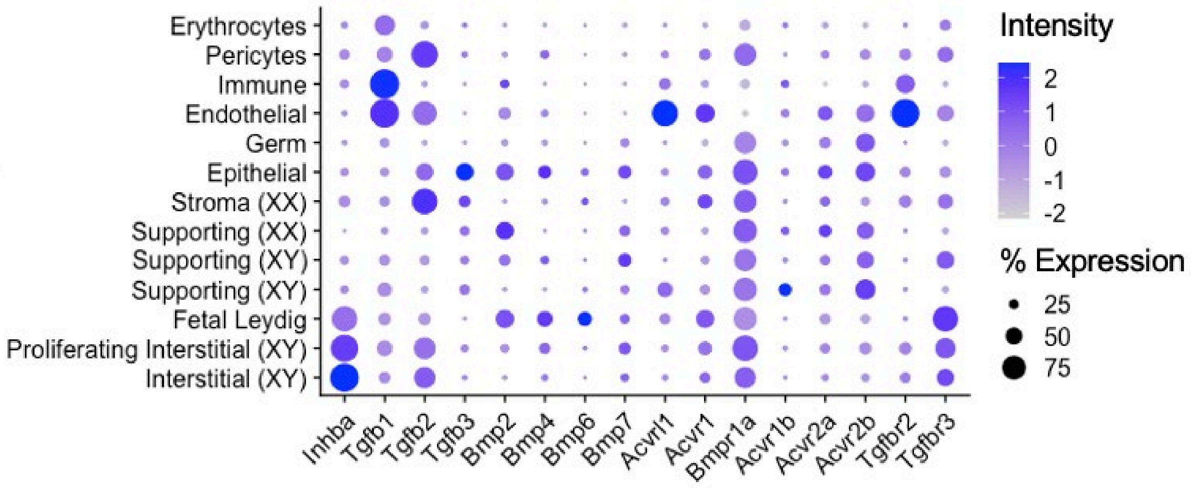


B

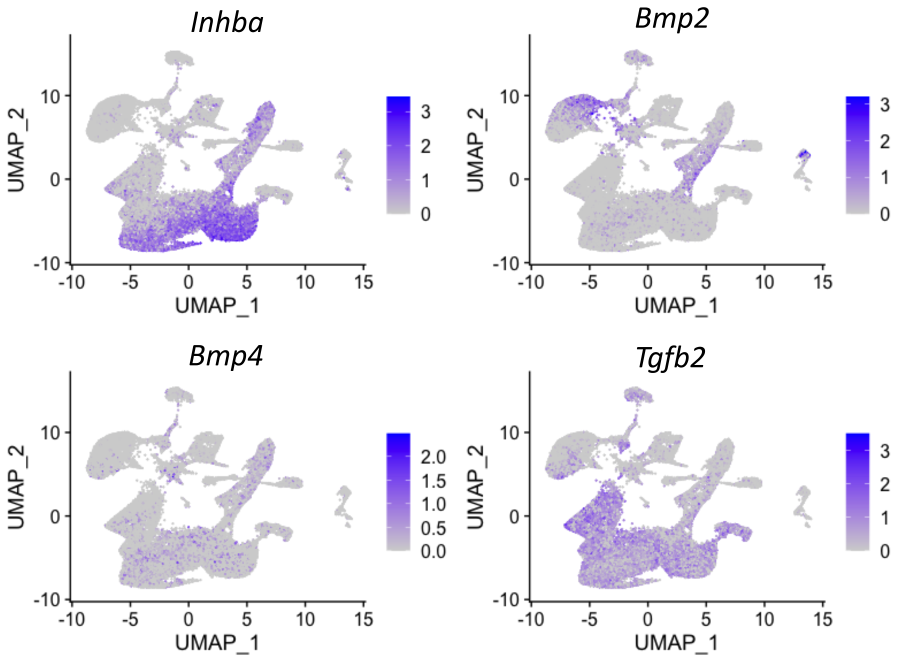


Supplemental Figure 4: Single cell analysis of control XX (ovary), control XY (testis) and dKO XY (ovotestis) at E15.5. (A) Dot plot of the gene expression of TGF-beta ligands and receptors in the different clusters. (B) UMAP distribution of the gene expression of the TGF-beta ligands: *Inhba, Bmp2, Bmp4 and Tgfb2.* N= 2 independent biological replicates per genotype.

**Supplemental Figure 5**

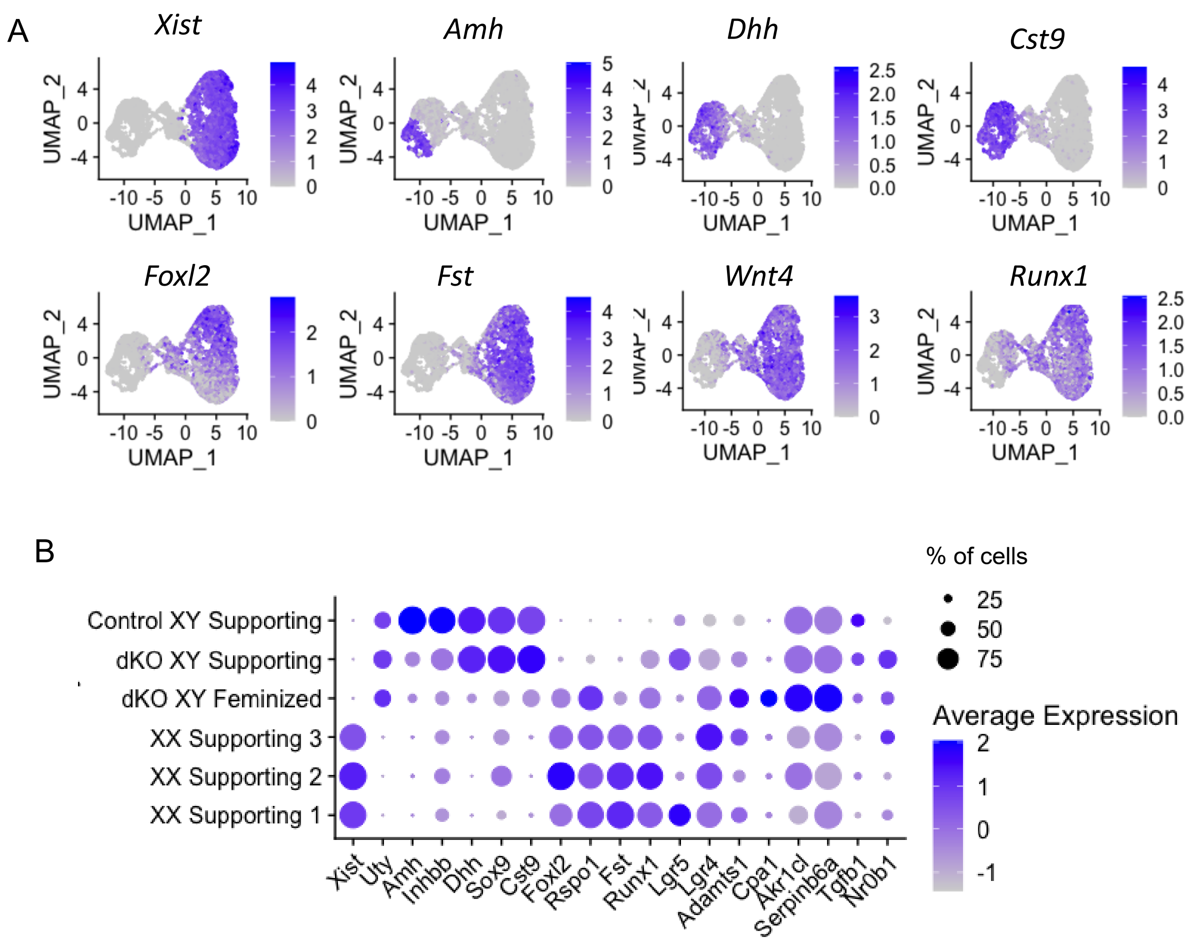


Supplemental Figure 5: Clustering of the supporting cells from control XY, dKO XY, and control XX gonads at E15.5. (A) Distribution on the UMAP of the female cell marker *Xist,* specific male supporting cell gene expression (*Amh, Dhh, Cst9)* and female supporting cell markers (*FoxL2, Rspo1, Fst, Runx1)*. (B) Dot plots of genes used to identify each cluster. N= 2 biological replicates per group.

**Supplemental Figure 6**

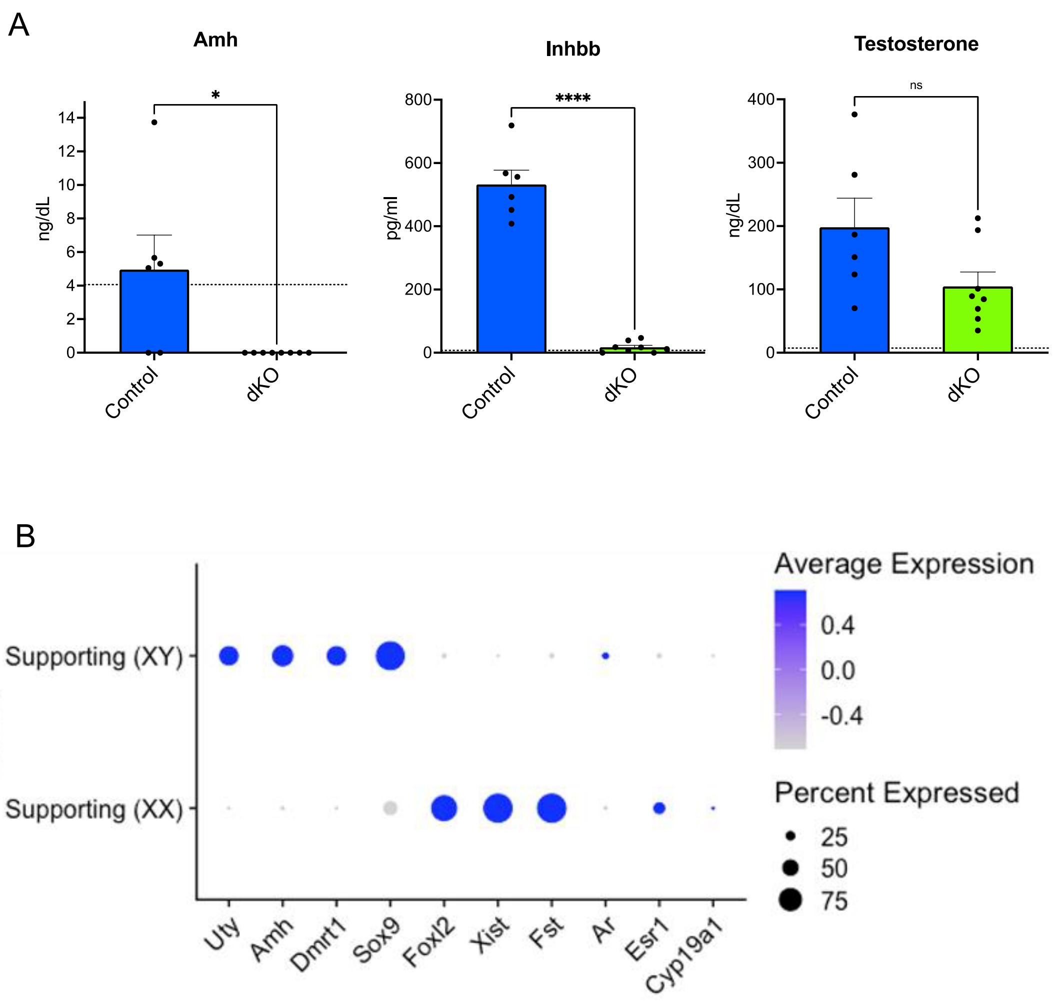


Supplemental Figure 6: Serum levels of AMH, activin B, and testosterone in control XY (n=6) and dKO XY (n=8) adult mice (Source data are provided as a Source Data file; two-tailed T-test, * p= 0.01, **** p<0.0001) Bars depict average +/- SE. The dotted line shows the detection limit of the assay. (B) Dot plots of genes expressed in XY and XX supporting cells based on the single cell RNAseq dataset.

**Supplemental Figure 7**

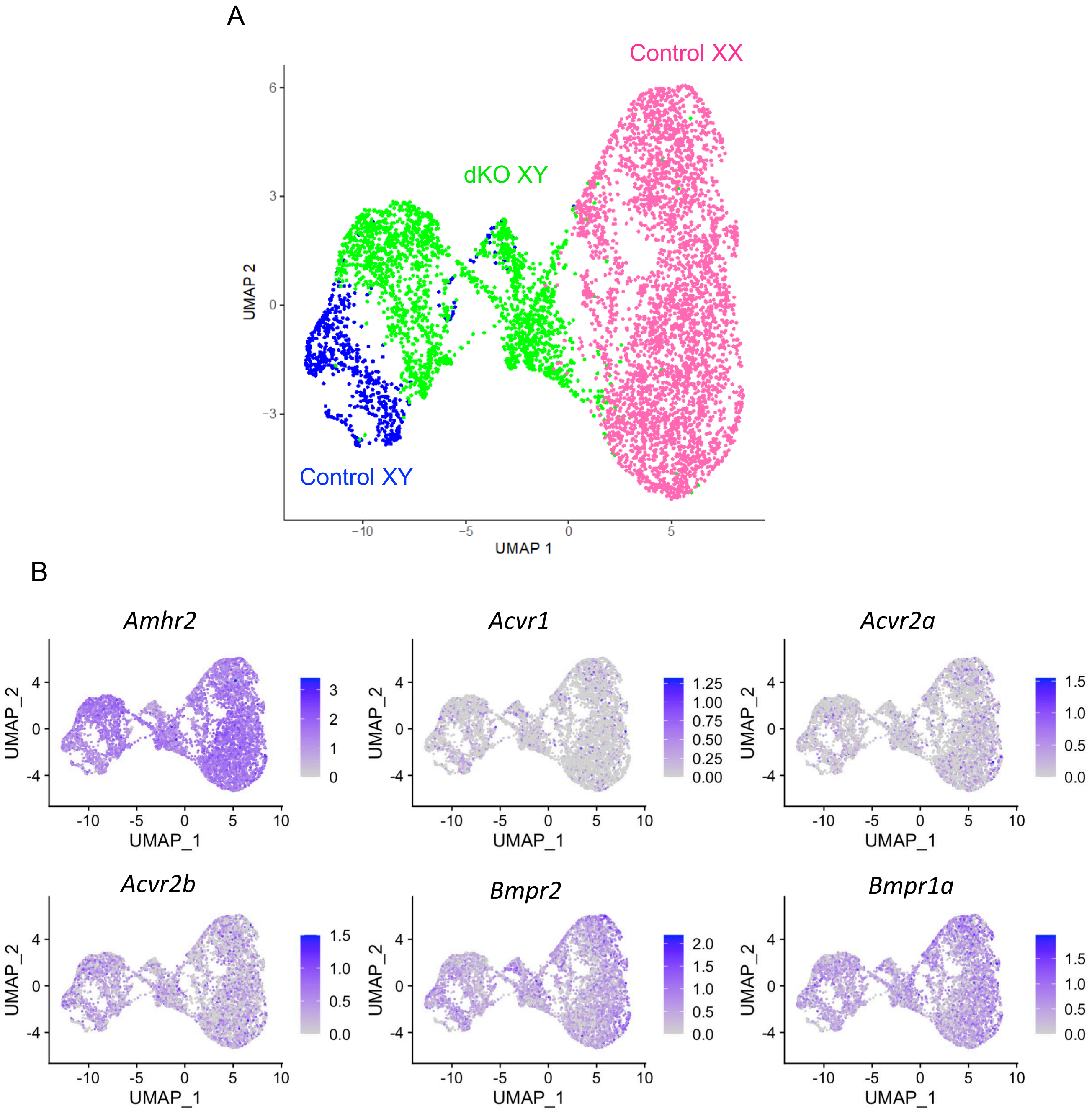


Supplemental Figure 7: (A) Single cell mRNA sequencing analysis and clustering of the supporting cells based on their genotypes: control XY (blue), dKO XY (green), and control XX (pink) gonads at E15.5. (B) Expression of receptors for AMH (*Amhr2, Bmpr2, Bmpr1a*) and activins (*Acvr1, Acvr2a, Acrr2b*) over the UMAP(A).
