# Supplementary material for: Somatic cell fate maintenance in mouse fetal testes via autocrine/paracrine action of AMH and activin B
Source: Nat Commun. 2022 Jul 15;13:4130. doi: 10.1038/s41467-022-31486-y (PMC9287316; doi:10.1038/s41467-022-31486-y)
Supplement: Supplementary file 2 — Reporting Summary [file 41467_2022_31486_MOESM2_ESM.pdf]

nature portfolio

Corresponding author(s): Dr. Humphrey Yao

Last updated by author(s): 2022/05/13

Reporting Summary

Nature Portfolio wishes to improve the reproducibility of the work that we publish. This form provides structure for consistency and transparency in reporting. For further information on Nature Portfolio policies, see our Editorial Policies and the Editorial Policy Checklist.

Statistics

For all statistical analyses, confirm that the following items are present in the figure legend, table legend, main text, or Methods section.

n/a	Confirmed	
☐	☒	The exact sample size (*n*) for each experimental group/condition, given as a discrete number and unit of measurement
☐	☒	A statement on whether measurements were taken from distinct samples or whether the same sample was measured repeatedly
☐	☒	The statistical test(s) used AND whether they are one- or two-sided<br>*Only common tests should be described solely by name; describe more complex techniques in the Methods section.*
☒	☐	A description of all covariates tested
☐	☒	A description of any assumptions or corrections, such as tests of normality and adjustment for multiple comparisons
☐	☒	A full description of the statistical parameters including central tendency (e.g. means) or other basic estimates (e.g. regression coefficient) AND variation (e.g. standard deviation) or associated estimates of uncertainty (e.g. confidence intervals)
☐	☒	For null hypothesis testing, the test statistic (e.g. *F*, *t*, *r*) with confidence intervals, effect sizes, degrees of freedom and *P* value noted<br>*Give P values as exact values whenever suitable.*
☒	☐	For Bayesian analysis, information on the choice of priors and Markov chain Monte Carlo settings
☒	☐	For hierarchical and complex designs, identification of the appropriate level for tests and full reporting of outcomes
☒	☐	Estimates of effect sizes (e.g. Cohen's *d*, Pearson's *r*), indicating how they were calculated

Our web collection on statistics for biologists contains articles on many of the points above.

Software and code

Policy information about availability of computer code

Data collection	Microarray data was obtained using the GeneChip® Command Console Software (AGCC; Version 3.2) and Expression Console (Version 1.2). Images were collected using Leica Application Suite X (LAS X v. 3.5.5.19976).
Data analysis	For the microarray, gene expression analyses were conducted with Partek software (Partek Genomics Suite v 7.19.1125, St. Louis, Missouri) using a one-way ANOVA comparing the RMA normalized log2 intensities. Prism (GraphPad Software v 9.3.0) was used to plot the log2 intensity data. Cell Ranger (v3.0) was utilized for count, alignment, filtering, cell barcode and UMI counting. Barcode swapping correction was performed. FASTQ files of the corrected cell count matrixes were generated. The data was analyzed using Seurat package (v.3.2.2) on RStudio (v. 1.3.1073). Seurat objects of each biological replicates were created (min.cells = 10, min.features = 400) and each biological replicate was combined using the "merge" function. A subset of the data was created using the following cutoffs: nFeature_RNA > 2500 & nFeature_RNA < 9000 & percent.mt < 25 & percent.Hbb < 20. The data was then normalized using the "NormalizeData" function (scale.factor = 10000). A linear model was applied to the data using the "Scaledata" command. PCA was run on the scaled data and visualization was done using UMAP with dims= 20. Differentially expressed marker genes in each cluster were found using FindAllMarkers function based on the Wilcoxon rank-sum test. luster identification was done according to established cell markers. Reclustering of male and female supporting cells as well as the feminized cluster was done using the "Subset" command, with data normalized and scaled, sorting out cells positive for Sycp3 and Tcf21 (germ and interstitial cell markers respectively). PCA was run on the scaled data and visualization was done using UMAP with dims= 8. A Seurat object from the reclustered cells was created and loaded into Monocle3 for pseudotime analysis to determine differentiation trajectory using Control XY supporting cells as time 0 (T0) in pseudotime. CellPhoneDB (https://www.cellphonedb.org), was used to predict cell-cell communication based on ligand-receptor interactions. Normalized counts were extracted from the Seurat object. Significant ligand-receptor pairs were called independently for wildtype males and dKO Germ, Supporting and Leydig cells respectively. The dotblot function in CellphoneDB was used to plot the data.

For manuscripts utilizing custom algorithms or software that are central to the research but not yet described in published literature, software must be made available to editors and reviewers. We strongly encourage code deposition in a community repository (e.g. GitHub). See the Nature Portfolio guidelines for submitting code & software for further information.

Data

Policy information about availability of data

All manuscripts must include a data availability statement. This statement should provide the following information, where applicable:

- Accession codes, unique identifiers, or web links for publicly available datasets
- A description of any restrictions on data availability
- For clinical datasets or third party data, please ensure that the statement adheres to our policy

The GEO accession numbers for the data sets are: E12.5 microarray GSE196826; E15.5 microarray GSE196841 and E15.5 scRNAseq GSE196973. The data sets used for the CellphoneDB analysis are listed in https://www.cellphonedb.org/downloads. Code used is supplied under supplemental information.

Field-specific reporting

Please select the one below that is the best fit for your research. If you are not sure, read the appropriate sections before making your selection.

[x] Life sciences [ ] Behavioural & social sciences [ ] Ecological, evolutionary & environmental sciences

For a reference copy of the document with all sections, see nature.com/documents/nr-reporting-summary-flat.pdf

Life sciences study design

All studies must disclose on these points even when the disclosure is negative.

Sample size	All experiments using mouse samples were perform on at least 4 independent biological replicates, except for the scRNA seq experiment that used 2 biological replicates. The number of biological replicates were determined based on accessibility of the tissues and the minimum number required to perform statistical analysis.
Data exclusions	No data were excluded from the analysis
Replication	Immunoflourescent experiments were repeated at least 4 times using independent biological replicates with successful replication. Microarray analysis were performed independently at each timepoint which included n=4 and n=5 independent biological replicates for the E12.5 and E15.5 timepoints respectively. Expression of key genes was recapitulated in the scRNA seq analysis data. Experiments were replicated successfully.
Randomization	Biological samples were allocated into experimental groups based on genotype.
Blinding	Blinding was not performed in this study because of the low availability of tissues from double KO animals.

Reporting for specific materials, systems and methods

We require information from authors about some types of materials, experimental systems and methods used in many studies. Here, indicate whether each material, system or method listed is relevant to your study. If you are not sure if a list item applies to your research, read the appropriate section before selecting a response.

Materials & experimental systems

n/a	Involved in the study	
[ ]	[x]	Antibodies
[x]	[ ]	Eukaryotic cell lines
[x]	[ ]	Palaeontology and archaeology
[ ]	[x]	Animals and other organisms
[x]	[ ]	Human research participants
[x]	[ ]	Clinical data
[x]	[ ]	Dual use research of concern

Methods

n/a	Involved in the study	
[x]	[ ]	ChIP-seq
[x]	[ ]	Flow cytometry
[x]	[ ]	MRI-based neuroimaging

Antibodies

Antibodies used	Anti-DMRT1 (1:500; kindly provided by David Zarkower from University of Minnesota, reported in Raymond et al,2000, PMC316999), FOXL2 (1:200, Novus Biologicals cat. #NB100-1277), LAMININ (1:300; Sigma #L9393), GCNA1 (1:1000, Abcam # ab82527), SYCP3 (1:300, Abcam # ab15093), SOX9 (1:300, TransGenic #KO608), NR2F2 (1:300, R&D Systems # PP-H7147-00) and AMH (1:500, Santa Cruz # sc-6886). Secondary antibodies were used at 1:200 dilution (Invitrogen/Life Technology): Donkey anti-rabbit Alexa 488 (A21206), Donkey anti-mouse Alexa 568 (A10037), Donkey anti-rat Alexa 594 (A21209), Donkey anti-goat Alexa 647 (A21447).
Validation	These antibodies have been previously optimized in Raymond et al. 2000 PMID: 11040213 (Anti-DMRT1), Nicol et al. 2018 PMID:

30212841 (Anti-FOXL2, Anti-SOX9, Anti-AMH) and Nicol et al. 2019 PMID: 31712577 (Anti-LAMININ, Anti-GCNA1, Anti-NR2F2, Anti SYCP3).

Animals and other organisms

Policy information about studies involving animals; ARRIVE guidelines recommended for reporting animal research

Laboratory animals	Control and double knockout of Amh and Inhbb mice were generated from crossing the single heterozygous Amh -/+ (Jax 002188) and Inhbb +/- (Jax 002442). Female mice were timed-mated, the day of detection of vaginal plug was considered embryonic day or E0.5. Tissues were collected at E12.5, E15.5 and adult. Gonads from E13.5 C57BL6/J (Jax 000664) mouse embryos were collected in the same manner for transplant experiments.
Wild animals	No wild animals were used in this study
Field-collected samples	No field collected samples were used in this study.
Ethics oversight	All mouse procedures were approved by the National Institutes of Health Animals Care and Use Committee, and were performed in accordance with an approved National Institute of Environmental Health Sciences animal study proposal.

Note that full information on the approval of the study protocol must also be provided in the manuscript.
